# Injurious mechanical ventilation causes kidney apoptosis and dysfunction during sepsis but not after intra-tracheal acid instillation: an experimental study

**DOI:** 10.1186/1471-2369-15-126

**Published:** 2014-07-29

**Authors:** Jan Willem Kuiper, AB Johan Groeneveld, Jack J Haitsma, Lonneke Smeding, Mark PV Begieneman, Serge Jothy, Rosanna Vaschetto, Frans B Plötz

**Affiliations:** 1 Department of Paediatric Intensive Care, Erasmus MC – Sophia Children’s Hospital, Dr. Molewaterplein 60, 3015 GJ Rotterdam, The Netherlands; 2 The Keenan Research Centre at the Li Ka Shing Knowledge Institute of St. Michael’s Hospital and Interdepartmental Division of Critical Care Medicine, University of Toronto, Toronto, Ontario, Canada; 3 Department of Intensive Care, Erasmus Medical Center, Rotterdam, The Netherlands; 4 Department of Pediatrics and Pediatric Intensive Care, VU Medical Center, Amsterdam. Institute for Cardiovascular Research, VU University Medical Center, Amsterdam, The Netherlands; 5 Department of Pathology, VU University Medical Center, Amsterdam, The Netherlands; 6 Department of Laboratory Medicine, St. Michael’s Hospital, University of Toronto, Toronto, Ontario, Canada; 7 Departments of Anesthesiology and Critical Care Medicine, Università del Piemonte Orientale “A. Avogadro” Alessandria-Novara-Vercelli, Viale Teresa Michel, 11, Alessandria, Italy; 8 Department of Clinical and Experimental Medicine, Università del Piemonte Orientale “A. Avogadro” Alessandria-Novara-Vercelli, Viale Teresa Michel, 11, Alessandria, Italy; 9 Department of Pediatrics, Tergooiziekenhuizen, Blaricum, The Netherlands

**Keywords:** Acute kidney injury, Acute respiratory distress syndrome, Apoptosis, Mechanical ventilation, Sepsis

## Abstract

**Background:**

Intratracheal aspiration and sepsis are leading causes of acute lung injury that frequently necessitate mechanical ventilation (MV), which may aggravate lung injury thereby potentially increasing the risk of acute kidney injury (AKI). We compared the effects of ventilation strategies and underlying conditions on the development of AKI.

**Methods:**

Spraque Dawley rats were challenged by intratracheal acid instillation or 24 h of abdominal sepsis, followed by MV with a low tidal volume (LV_T_) and 5 cm H_2_O positive end-expiratory pressure (PEEP) or a high tidal volume (HV_T_) and no PEEP, which is known to cause more lung injury after acid instillation than in sepsis. Rats were ventilated for 4 hrs and kidney function and plasma mediator levels were measured. Kidney injury was assessed by microscopy; apoptosis was quantified by TUNEL staining.

**Results:**

During sepsis, but not after acid instillation, MV with HV_T_ caused more renal apoptosis than MV with LV_T_. Increased plasma active plasminogen activator inhibitor-1 correlated to kidney apoptosis in the cortex and medulla. Increased apoptosis after HV_T_ ventilation during sepsis was associated with a 40% decrease in creatinine clearance.

**Conclusions:**

AKI is more likely to develop after MV induced lung injury during an indirect (as in sepsis) than after a direct (as after intra-tracheal instillation) insult to the lungs, since it induces kidney apoptosis during sepsis but not after acid instillation, opposite to the lung injury it caused. Our findings thus suggest using protective ventilatory strategies in human sepsis, even in the absence of overt lung injury, to protect the kidney.

## Background

Both acute lung injury (either direct or indirect) and mechanical ventilation (MV) are important contributing factors for the development of acute kidney injury (AKI) in patients, but how they interact is unclear [1-9]. This is important since AKI is a common problem in critically ill patients and carries significant morbidity and mortality, so that identification of mechanisms or modifiable risk factors may help to understand and manage AKI for the benefit of patients [9,10].

The effect of injurious high tidal volume (HV_T_) versus non-injurious low tidal volume (LV_T_) MV may depend on the underlying condition, such as aspiration pneumonia or sepsis [11], and this may similarly translate into differences in susceptibility to AKI [12]. In humans, HV_T_ vs LV_T_ MV did not increase the incidence of AKI in patients without lung injury [13], but AKI was more common after HV_T_ than LV_T_ MV in patients with acute respiratory distress syndrome [14]. Animal models of intra-tracheal acid and lipopolysaccharide (LPS) instillation demonstrate that HV_T_ MV is associated with increased kidney interleukin-6, vascular endothelial growth factor levels, apoptosis and necrosis compared to LV_T_ MV [15-18]. However, these observations have not been confirmed by others. For example, Hoag et al. did not find an effect of acid instillation followed by HV_T_ MV on kidney apoptosis or kidney function [12]. In a sepsis model, O’Mahoney et al. showed increased pulmonary cytokines and pulmonary permeability and increased plasma creatinine levels and protein accumulation in collecting tubules after LV_T_ MV following intraperitoneal LPS injection, but not after MV or LPS alone [19]. Currently, known effects of MV on AKI during acute lung injury remain inconclusive and, to date, no study compared the effects of MV after either direct or indirect lung injury on the development of AKI. Otherwise, a growing body of evidence suggests that apoptosis plays a key role, particularly in inflammatory conditions, in the pathogenesis of AKI, and induction highly depends on underlying conditions [6,20-22].

Therefore, in the current study we set out to investigate whether the effects of HV_T_ MV on kidney apoptosis and function are differentially affected by the underlying acute lung injury; i.e. following either direct or indirect lung injury. We hypothesized that increased ventilator-induced lung injury after intra-tracheal acid instillation (direct lung injury) results in increased kidney apoptosis and decreased kidney function, as compared to less severe lung injury after cecal ligation and puncture (CLP)-induced sepsis (indirect lung injury). The present study uses the animals from our previous study and expands on this study by investigating the effect of different MV strategies on the development of kidney injury, apoptosis and dysfunction [11].

## Methods

### Animal preparation

Ethical approval was obtained from the Institutional Animal Care Committee of St Michael’s Hospital and animals were treated according to the Canadian national guidelines. A completed Animal Research: Reporting In Vivo Experiments checklist has been submitted. Animals were housed in the animal care facility of St Michael’s Hospital with unlimited access to food and water. The animals used in the present study were those studied previously [11]. We will report new data on kidney histology, apoptosis and kidney function. Adult, male Sprague Dawley rats (Charles Rivers, St Constan, QC, Canada) weighing 290 - 320 g were anesthetized with xylazine (Bayer, Toronto, ON, Canada) 10 mg/kg and ketamine (Bimeda-MTC, Cambridge, ON, Canada) 100 mg/kg given intraperitoneally. Anaesthesia was maintained by intravenous xylazine 1 mg/kg/h, ketamine 20 mg/kg/h; muscle relaxation was achieved by intravenous pancuronium bromide (Sabex Inc, QC, Canada) 0.6 mg/kg/h. During surgical procedures and ventilation, rats were supine on a heating pad and body temperature was maintained at 37°C. For blood sampling, fluid infusion and arterial blood pressure measurements, catheters were inserted into the right carotid artery and tail vein before stabilization. The arterial catheter was connected to a pressure transducer for continuous measurement of arterial blood pressure. During MV all animals received a continuous infusion of normal saline to maintain mean arterial blood pressure >60 mmHg, and for patency of intravenous lines. Additionally, in these animals the bladder was catheterized using a transabdominal approach for collection of urine.

Intratracheal acid instillation, as a model for aspiration of gastric content, primarily targets the pulmonary epithelium. This model followed by MV is used to reproduce clinically relevant scenarios [23]. A pilot study was undertaken to establish the acid instillation protocol. Briefly, after anaesthesia and tracheotomy, a 14G canula was inserted into the trachea and connected to a ventilator (Servo 300, Siemens, Munich, Germany); set to deliver a tidal volume (V_T_) of 6 mL/kg and a positive end-expiratory pressure (PEEP) of 5 cm H_2_O. One animal was ventilated per ventilator per experiment. Arterial and venous catheters were inserted and hydrochloric acid (HCl, pH 2.0), 1 ml/kg, was rapidly instilled intratracheally at baseline using an aerosolizer (PennCentury Inc, Philadelphia, PA, USA). Instillation was followed by a recruitment manoeuvre (increase in PEEP to 25 cm H_2_O for 5 breaths). Rats were subsequently stabilized for 10 minutes and then randomized. Control rats received acid instillation alone after which they were sacrificed after the recruitment manoeuvre. Rats did not survive acid instillation without subsequent MV due to technical and ethical limitations; inclusion of time-matched controls was therefore impossible. The mortality rate of acid instillation was 13% before randomisation.

CLP-induced polymicrobial sepsis is one of the best and widely used animal models for the study of sepsis and organ damage, including lung and kidney damage.[23] With the animal spontaneously breathing 40% oxygen, a laparotomy through a midline incision using an aseptic technique was performed. The coecum was ligated just below the ileocecal valve with 3–0 silk ligature, so that intestinal continuity was maintained. Using a 14-Gauge needle, the coecum was perforated in two locations, 1 cm apart, on the antimesenteric surface of the coecum, and the coecum was gently compressed until faeces were extruded. The bowel was then returned to the abdomen and the incision was closed using 4–0 silk ligature for both the muscle layer and skin. Subsequently, rats received 30 mL/kg 0.9% saline in the scruff of the neck and buprenorphine 30 μg/kg subcutaneously (Schering-Plough, Hertfordshire, UK). The rats breathed 40% oxygen until recovery from anaesthesia, and then were placed back in a cage with free access to food and water. Eight hours after surgery, rats received a 30 mL/kg 0.9% saline bolus i.p. Mortality rate of the model prior to randomisation was 6%. Twenty-four hrs after the induction of sepsis, rats were anaesthetized and tracheotomy was performed, with a canula (14 gauge) inserted into the trachea. Rats were connected to a ventilator; arterial and venous catheters were inserted followed by a 10-minute stabilization period with ventilation using V_T_ 6 mL/kg and PEEP 5 cm H_2_O.

### Experimental protocol

**Figure 1:**
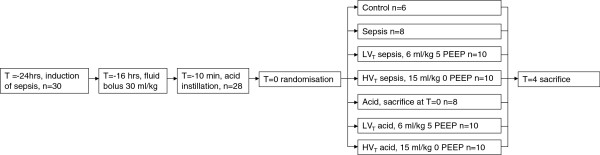
**Timeline of the experiment.** Septic rats and rats after acid instillation were mechanically ventilated for 4 hrs after which blood was drawn, the rats were sacrificed and the organs harvested. See text for further details. HV_T_: high tidal volume, LVT: low tidal volume, PEEP: positive end-expiratory pressure.

After stabilization, rats were randomly allocated to one of 4 groups: MV with either a low V_T_ (6 mL/kg) and PEEP 5 cm H_2_O (n = 10 per group) (LV_T_ acid and LV_T_ sepsis) or a high V_T_ (15 mL/kg), no PEEP (n = 10 per group) (HV_T_ acid and HV_T_ sepsis). These strategies are widely used and advocated as safe or mildly lung-injurious in rats [17,24-26]. Additionally, a V_T_ of more than 15 ml/kg is necessary to injure the lungs during sepsis in rats, as shown before [26]. Eight rats were immediately sacrificed after acid instillation (acid). Ten septic rats served as non-ventilated septic controls (sepsis) and were sacrificed 28 hr after induction of sepsis. Six healthy rats served as healthy controls (control) (Figure 1). Inspiratory/expiratory ratio was 1:2. Normocapnia was maintained by adjusting respiratory rate. The fraction of inspired oxygen was set at 0.4 and increased when necessary in ventilated groups. At the end of the experiment a blood sample was taken and animals were sacrificed with an overdose of ketamine/xylazine.

Rats were ventilated in the laboratory for 4 hrs during which blood pressure and heart rate were measured continuously. Arterial blood samples were taken 30 min after randomization and every hour for blood gas analysis (Ciba Corning Model 248 blood gas analyser, Corning Medical, Medfield, MA, USA). For each blood sample a volume of maximum 100 μl was necessary. An equal amount of normal saline was administered intra-venously to compensate for the fluid loss. After 3 hrs of mechanical ventilation, bladders were emptied and urine samples were collected during the last hour of the experimental protocol. At the end of the experiment a blood sample was taken and animals were sacrificed with an overdose of intravenous anaesthesia. Non ventilated controls were sacrificed 28 hrs after induction of sepsis. Lungs and kidneys were harvested for histological examination. Creatinine clearance was calculated using the formula U_Cr_xV/P_Cr_, where U_Cr_ represents the urine creatinine concentration (mg/ml), V is the urine flow (ml/min) and P_Cr_ is the plasma creatinine concentration. Technically, it was not possible to collect urine in spontaneously breathing control animals without subjecting the rats to anaesthesia and subsequent mechanical ventilation. Mediators were measured in plasma as described and presented previously [11].

### Histology

A pathologist, blinded as to the experimental history of the specimens, performed a quantitative morphometric analysis of kidney injury using a scoring system that included tubular dilatation, presence of intra-tubular debris, vacuolization of tubular epithelium cells and loss of brush border membrane integrity. Lung injury was assessed as described previously and some data were reiterated for the sake of clarity [11]. Briefly, a quantitative morphometric analysis of alveolar collapse, perivascular and alveolar haemorrhage, perivascular oedema, vascular congestion, alveolar polymorphonuclear leukocytes, alveolar oedema and macrophages was performed blindly by a pathologist (scores potentially ranged from 0 to 24).

### Apoptosis (TUNEL assay)

Apoptotic cells were detected using a terminal deoxynucleotidyl transferase dUTP nick end labelling (TUNEL) assay for in-situ end labelling, adapted to an automated in-situ hybridization instrument (Discovery™ Ventana Medical Systems, Inc.Tuscon, AZ. USA). As per Discovery protocol, the instrument used 5 μm thick deparaffinised tissue sections mounted on positive charged glass slides, with subsequent digestion with Protease I (Ventana Medical Systems, AZ. USA) digestion for 12 minutes. The assay uses recombinant terminal deoxynucleotidyl transferase (Tdt) (Invitrogen Corporation, CA, USA.) for adding homo-polymer tails to the 3’ ends of cleaved DNA, characteristic in cells undergoing programmed cell death. Biotin 16-dUTP (Roche Diagnostics, Basel Switzerland) was the labelled nucleotide used for this reaction. Colorimetric visualization using avidin-horse radish peroxidase and 3,3′-diaminobenzidine detection method was performed. Cells were counterstained with haematoxylin to facilitate cell counting. Twelve randomly chosen fields of each section (72 fields for each group) were counted in a blinded fashion. An apoptotic index was calculated [100% × TUNEL-positive cells)/(total number of cells)].

### Statistical analysis

Data are expressed as mean ± standard error of the mean. When data were not normally distributed according to a Kolmogorov-Smirnov test (P > 0.05), data were ranked before analysis. The effects of MV in each model were tested using univariate analysis of variance and longitudinal data were compared using generalized estimating equations designed for the analysis of repeated measurements. Post hoc testing was performed according to Bonferroni. Using these tests the effects of the model were analysed for each parameter and these comparisons are described by acid instillation or sepsis. Subsequently the effects of MV were analysed and finally the model-dependent effects of MV were analysed by determining the interaction between model and MV for each parameter. Depending on normality of the data Pearson’s correlation or Spearman’s Rho were calculated. A value of p < 0.05 was considered statistically significant, we report exact p-values unless p < 0.001. All analyses were performed using SPSS 21.0 statistical software (SPSS Inc., Chicago, IL, USA).

## Results

### Lung injury

Lung injury was more severe after MV following acid instillation compared to sepsis as reported [11]. Lung wet/dry weight ratio after 4 hrs of MV was 8.1 ± 0.3 for HV_T_ after acid instillation as compared to 5.6 ± 0.1, 5.0 ± 0.1 and 5.5 ± 0.1 for LV_T_ after acid instillation or sepsis and HV_T_ during sepsis, respectively. Acid instillation increased the ratio as compared to sepsis (P < 0.001) and HV_T_ increased wet/dry weight ratio after acid instillation but not after sepsis (P < 0.001) [11]. Histological lung injury score after 4 hrs of MV was 11.2 ± 0.9 for HV_T_ acid as compared to 7.4 ± 0.6, 4.3 ± 0.5 and 5.3 ± 0.3 for LV_T_ acid, LV_T_ sepsis and HV_T_ sepsis, respectively. Acid instillation increased lung injury score compared to sepsis (P < 0.001) and HV_T_ increased lung injury score after acid instillation, but not after sepsis (P = 0.04) [11].

### Kidney histology and apoptosis

**Figure 2:**
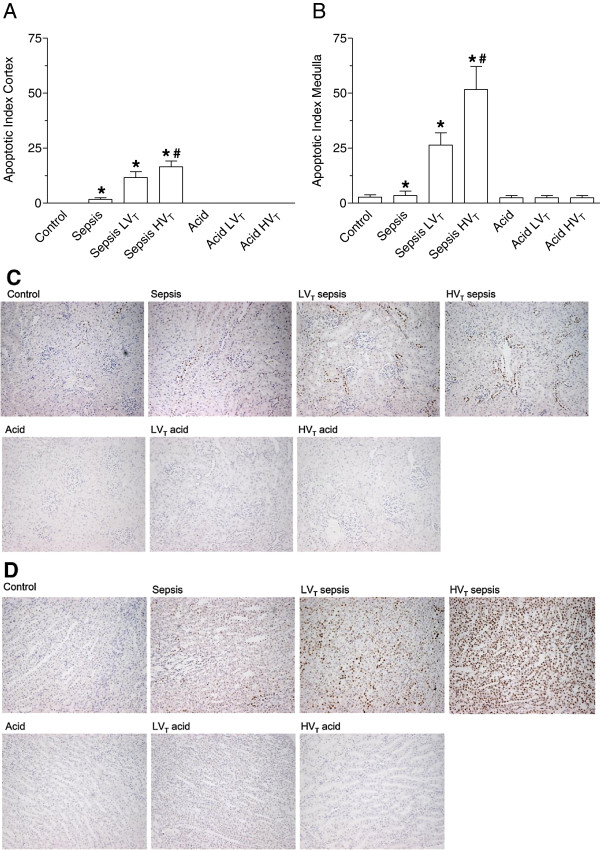
**Kidney apoptosis after mechanical ventilation. A** and **B** show apoptotic indexes of renal cortex and medulla. Rats were ventilated for 4 hrs with different ventilatory strategies during sepsis or after intratracheal acid instillation. Healthy rats, rats subjected to sepsis or acid instillation alone served as controls. During sepsis apoptosis was increased compared to acid instillation. MV increased apoptosis and as indicated by an interaction between model and MV, MV with HV_T_ increased apoptosis during sepsis, but not during acid instillation. **C** and **D** show representative photomicrographs of TUNEL stained apoptotic cells in renal cortex and medulla respectively (hematoxylin counterstain, magnification 400×). Apoptosis in cortex is most pronounced in endothelial cells after MV with HV_T_. LV_T_: mechanical ventilation with low tidal volume, HV_T_: mechanical ventilation with high tidal volume. *P < 0.001 compared to acid and control, ^#^P < 0.001 compared to LV_T_ sepsis and sepsis.

No differences in histological injury were observed between the groups (data not shown). In the kidney cortex and medulla apoptosis was observed after MV during sepsis, but not after MV after acid instillation (P < 0.001, Figure 2). Kidney cortical and medullary apoptosis also depended on the MV strategy (P < 0.001). As indicated by a significant interaction between model and MV strategy (P < 0.001), MV with HV_T_ caused increased apoptosis during sepsis but not after acid instillation (Figure 2).

### Mediators and apoptosis

We correlated plasma levels of interleukin-6, tumor necrosis factor-α, macrophage inflammatory protein-2, active plasminogen activator inhibitor-1 (aPAI-1) and soluble intercellular adhesion molecule-1 [11] to apoptosis in kidney medulla and cortex. Only plasma aPAI-1 levels correlated to apoptosis in cortex and medulla (Spearman’s Rho 0.51, P = 0.004 and 0.40, P = 0.03, respectively).

### Kidney function

**Figure 3:**
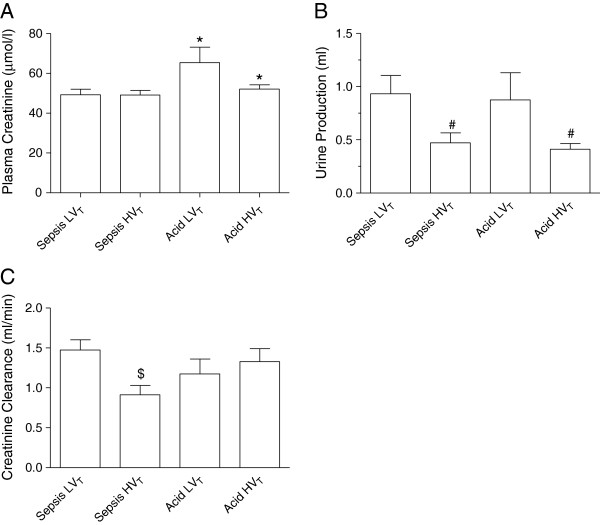
**Rats were ventilated for 4 hrs with different ventilatory strategies during sepsis or after intratracheal acid instillation.** Plasma creatinine levels were higher after acid instillation (n = 17) than after MV during sepsis (n = 20) **(A)**. HV_T_ decreased urine production as compared to LV_T_**(B)**. Indicated by a significant interaction, HV_T_ decreased creatinine clearance during sepsis (n = 20) but not after acid instillation (n = 17) **(C)**. LV_T_: mechanical ventilation with low tidal volume, HV_T_: mechanical ventilation with high tidal volume. *P = 0.040 as comapred to sepsis, ^#^P = 0.009 as compared to LV_T_, ^$^P = 0.016 as compared to acid HV_T_.

Plasma creatinine levels were higher during MV after acid instillation (n = 17/20) than during sepsis (n = 20/20, P = 0.040). No effects of MV strategy on plasma creatinine levels were observed (P = 0.53). The lack of an interaction between model and MV strategy indicate that there were no model dependent effects of MV strategy on plasma creatinine levels (P = 0.39, Figure 3A). Urine production did not differ between the acid and sepsis model (P = 0.47). HV_T_ decreased urine production as compared to LV_T_ (P = 0.009). There were no model dependent effects of MV with HV_T_ on urine production (P = 0.54, Figure 3B). Creatinine clearance did not differ between acid instillation (n = 17/20) and sepsis (n = 20/20, P = 0.74). Also, MV strategy did not affect creatinine clearance (P = 0.15). However, as indicated by an interaction (P = 0.016), HV_T_ decreased creatinine clearance during sepsis but not after acid instillation (Figure 3C).

## Discussion

The most important findings of our study are that the effects of injurious MV on kidney apoptosis depend on the underlying type of acute lung injury. However, in contrast to our hypothesis, minimally lung-injurious HV_T_ MV during sepsis caused kidney apoptosis, whereas HV_T_ MV after intratracheal acid instillation was associated with severe lung injury but less kidney apoptosis. Second, kidney apoptosis was associated with a greater than 40% decrease in creatinine clearance after HV_T_ as compared to LV_T_ ventilation.

We found that HV_T_ during sepsis, as opposed to sepsis alone, caused kidney apoptosis in the absence of relevant lung injury. For apoptosis to occur during sepsis alone, more than one hit may be required [17]. During sepsis systemic injury and inflammation occur which may increase the sensitivity of the kidney to apoptosis induced by ventilator-induced lung injury, whereas MV after intratracheal acid instillation may only increase local pulmonary injury and inflammation.

The observed MV induced kidney apoptosis may be explained by several mechanisms proposed previously [4]. First, MV can induce kidney apoptosis by a direct effect of the systemic release of pulmonary produced toxic mediators [4,7]. We observed that only systemic aPAI-1 levels correlated with kidney apoptosis. In a rat model of pneumonia, MV with high tidal volume causes pro-coagulant changes and attenuated fibrinolysis in the lungs with alterations in systemic fibrin turnover [27]. Although aPAI-1 was increased in the lung, aPAI-1 was not measured in the systemic circulation [27]. Several effects of aPAI-1 on the development of kidney injury have been described [7]. In-vitro, aPAI-1 can induce apoptosis in endothelial cells [28]. Studies in animal models have shown that aPAI-1 messenger RNA levels in the kidney were elevated after CLP in a model of sepsis-induced acute kidney injury [29]. Also in humans, aPAI-1 levels measured on days 0, 1 and 3 during the ARDS network trial were independently associated with AKI as measured by increased serum creatinine levels compared to baseline [30]. In contrast, a recent study found that baseline aPAI-1 levels were not predictive of the need for renal replacement therapy but this study did not report on the incidence of AKI without the need for renal replacement therapy [30]. Although the positive correlation of aPAI-1 and renal apoptosis may suggest a pivotal role for aPAI-1 in the development of AKI during sepsis and MV several issues remain unanswered. In contrast to our hypothesis we expected that more severe ventilator-induced lung injury would be associated with higher systemic levels of mediators, and consequently more apoptosis. Since this did not happen it remains questionable if the lungs are indeed the source of aPAI-1. In this regard aPAI-1, rather than being the cause of the increased kidney apoptosis, could be produced directly by kidney tubular cell in response to the ischemic damage caused by dysregulation of kidney vasoactive mechanism induced by sepsis and worsened theoretically by mechanical ventilation [31,32]. The production of aPAI-1 by tubular cells is increased in hypoxic condition and aPAI-1 is known to exert direct and indirect apoptotic effect [31,32].

Second, kidney apoptosis can be induced through an effect on both global and regional renal blood flow. Global differences in renal blood flow can be caused by hypoxia and/or hypercapnia. Therefore, we kept P_a_O_2_ and P_a_CO_2_ within normal limits in this study to avoid effects of MV on gas exchange with subsequent effects on renal blood flow. Also, mean arterial pressure was kept above 60 mmHg and was similar between the groups. However, the apoptosis we observed was unevenly distributed in the kidney, the apoptotic index was higher in the medulla compared to the cortex. The higher apoptotic index in the medulla suggests, despite similar mean arterial blood pressures, regional differences in renal blood flow. This indicates that HV_T_ MV during sepsis may affect local perfusion in the kidney and, as mentioned before, as a result in local production of aPAI-1.

We showed previously that MV with HV_T_ in a rat model of pneumonia was associated with impaired kidney endothelium-dependent vasodilatation [24]. Whether these changes may cause regional differences in perfusion is unknown. However, the impaired vasodilatation was attenuated after administration of a poly (adenosine diphosphate-ribose) polymerase (PARP) inhibitor [24]. Two studies by the same group showed that during sepsis vasodilatation occurs with an increase in renal blood flow but with decreased creatinine clearance [33,34]. These findings may be explained by effects on kidney afferent and efferent arterioles leading to decreased glomerular capillary pressure [35]. Damaging effects of cytokines, possibly released following increased and sustained sympathetic nerve activity, have been suggested [36]. However, the exact mechanisms of arteriolar dysfunction remain unknown, and possibly, impaired fibrinolysis by increased aPAI-1 levels leading to endothelial dysfunction plays a role [30].

There is increasing evidence for a pivotal role of apoptosis in AKI and septic AKI in humans [21,35]. Recently, a post-mortem study in patients with AKI associated septic shock demonstrated increased kidney tubular apoptosis [20], but these data have not been confirmed by others [8]. Additionally, genetic polymorphisms in the apoptosis regulatory protein BCL-2 gene protected against developing AKI during septic shock and MV [37]. In a murine model of septic kidney injury the level of kidney dysfunction directly correlated with apoptosis [22]. Although apoptosis can be reliably detected by TUNEL staining, different tests to detect apoptosis are usually performed to support the evidence of apoptosis obtained by TUNEL staining [38]. We confirmed the TUNEL data by various other methods previously [17]. In our study, MV induced kidney apoptosis was associated with a more than 40% decrease in creatinine clearance. Although creatinine can be actively excreted by the Lewis rat kidney, creatinine clearance is commonly used to evaluate kidney function [24,39]. Moreover endogenous creatinine clearance is strain specific [40]. In Wistar rats, from which Sprague Dawley rats were developed, craetinine clearance is similar to inulin clearance [40]. Plasma creatinine was higher in rats after acid instillation compared to sepsis. This difference is explained by one rat with an plasma creatinine level, substantially higher than the average level of plasma creatinine in rats subjected to MV after acid instillation.

The degree of sepsis in our model was relatively mild and of short duration prior to MV. This explains the absence of lung injury after sepsis and HV_T_. Although CLP-induced polymicrobial sepsis is one of the best and most widely used animal models for sepsis and organ injury the bacterial inoculum is unknown and severity may vary accordingly [23]. CLP-induced sepsis can also cause lung injury, mainly targeting the pulmonary endothelium [23]. Campos et al., reported a 24 hr mortality rate of 50% after sepsis, whereas we observed a 6% mortality rate [41] indicating a less severe sepsis with likely less endothelial damage in our study. The more severely injured pulmonary endothelium found by Campos et al., is highlighted by the increased wet-to-dry lung weight ratio [41]. The increased endothelial damage may have attracted more polymorphonuclear granulocyes, including neutrophils with subsequently more oxidative stress than in our study where the pulmonary endothelium was not damaged [11,41]. The additional effect of HV_T_ MV on lung injury in our study was limited, similar to others where they found that a V_T_ of more than 15 ml/kg was necessary to injure the lungs during sepsis [26].

In contrast to sepsis, MV following acid instillation in our study did not cause kidney apoptosis. Previous animal studies of acid instillation induced lung injury followed by injurious MV reported conflicting findings on kidney injury and apoptosis [12,15,18]. Imai et al. showed that after 8 hrs of MV in rabbits following intra-tracheal acid instillation HV_T_ (15–17 ml/kg) MV increased kidney epithelial cell apoptosis 6-fold compared to a LV_T_ MV [15]. After 4 hrs, this was not associated with increased plasma creatinine levels, but after 8 hrs, creatinine was higher after HV_T_. In contrast, Hoag et al., did not observe kidney apoptosis after 5 hrs of MV with HV_T_ (25 ml/kg) following acid instillation or sham treatment in dogs. Furthermore, in this study, various measurements of kidney function did not differ between the groups [12]. Hoag et al. suggested that in the study by Imai et al. the mean arterial pressure was maintained between 55–60 mmHg. This low mean arterial pressure may account for some of the alterations observed in plasma creatinine as a consequence of reduced renal blood flow, which was not measured [12]. Species differences and severity of lung injury may have affected the differences in outcome in these studies.

This study has some limitations. Since rat chest wall and lung mechanics differ from the human situation these results cannot be translated to the human situation directly. We used a V_T_ of 15 ml/kg with no PEEP as a proof of concept. These settings are not used in humans since they are associated with increased lung injury and death in patients with ARDS [14]. However, we observed increased renal apoptosis after HV_T_ in the absence of functional and histological lung injury. This suggests that during sepsis without lung injury MV with settings that do not directly injure the lung there may be effects on the kidney, especially since V_T_’s greater than 6 ml/kg are still used [42].

## Conclusions

We show that MV-induced kidney apoptosis depends on the underlying condition and primary type of lung injury. During sepsis, MV with HV_T_ did not cause overt lung injury, but increased kidney apoptosis as compared to LV_T_. Moreover, this was associated with decreased kidney function and increased aPAI-1 levels. Although intratracheal acid instillation caused more severe ventilator-induced lung injury, HV_T_ did not increase kidney apoptosis. Our findings thus suggest using protective ventilatory strategies in human sepsis, even in the absence of overt lung injury, to protect the kidney.

## Abbreviations

MV: Mechanical ventilation; AKI: Acute kidney injury; HV_T_: High tidal volume; LV_T_: Low tidal volume; LPS: Lipopolysaccharide; V_T_: Tidal volume; PEEP: Positive end expiratory pressure; U_Cr_: Urine creatinine concentration; V: Urine flow; P_Cr_: Plasma creatinine concentration; TUNEL: Terminal deoxynucleotidyl transferase dUTP nick end labelling; aPAI-1: Active plasminogen activator inhibitor-1.

## Competing interests

The authors declare that they have no competing interests.

## Authors’ contributions

JWK participated in the design of the study, performed the animal experiments and drafted the manuscript. ABJG conceived of the study, participated in the design of the study and helped to draft the manuscript. JJH participated in the design and coordination of the study and assisted with the animal experiments and with the drafting of the manuscript. LS was critical in the mediator assays and helped to draft the manuscript. MPVB was critical in the mediator assays and helped to draft the manuscript. SJ performed the histology studies and critically reviewed the manuscript. RV participated in the design and coordination and assisted with the animal experiments. FBP conceived of the study, participated in the design of the study and helped to draft the manuscript. All authors read and approved the final manuscript.

## Pre-publication history

The pre-publication history for this paper can be accessed here:

http://www.biomedcentral.com/1471-2369/15/126/prepub
